# Expression profiles of FABP4 and FABP5 in breast cancer: clinical implications and perspectives

**DOI:** 10.1007/s12672-025-02117-x

**Published:** 2025-03-19

**Authors:** Xingshan Jiang, Yiqin Xiong, Jianyu Yu, Anthony Avellino, Shanshan Liu, Xiaochun Han, Zhaohua Wang, Jonathan S. Shilyansky, Melissa A. Curry, Jiaqing Hao, Edward R. Sauter, Yi Huang, Sonia L. Sugg, Bing Li

**Affiliations:** 1 Department of Pathology, University of Iowa, 431 Newton Road, Iowa City, IA 52242 USA; 2 Holden Comprehensive Cancer Center, University of Iowa, Iowa City, IA USA; 3 Division of Cancer Prevention, NIH/NCI, Rockville, MD USA; 4 Department of Internal Medicine, University of Iowa, Iowa City, IA USA; 5 Department of Surgery, University of Iowa, Iowa City, IA USA

## Abstract

The incidence of breast cancer continues to rise each year despite significant advances in diagnosis and treatment. Obesity-associated dysregulated lipid metabolism is believed to contribute to the increasing risk of breast cancer. However, the mechanisms linking lipid dysregulation to breast cancer risk and progression remain to be determined. The family of fatty acid binding proteins (FABPs) evolves to facilitate lipid transport and metabolism. As the predominant isoforms of FABP members expressed in breast tissue, adipose FABP (A-FABP, also known as FABP4) and epithelial FABP (E-FABP, FABP5) have been shown to play critical roles in breast carcinogenesis. In this study, we collected surgical breast tissue samples from 96 women with different subtypes of breast cancer and comprehensively analyzed the expression pattens of FABP4 and FABP5. We found that distinct expression profiles of FABP4 and FABP5 were associated with their unique roles in breast cancer development. FABP4, mainly expressed in breast stroma, especially in adipose tissue, likely supported neighboring tumor cell lymphovascular invasion through secretion from adipocytes. In contrast, FABP5, primarily expressed in epithelial-derived tumor cells, could promote tumor metastasis by enhancing lipid metabolism. Thus, elevated levels of FABP4 and FABP5 may serve as poor prognostic markers for breast cancer. Inhibiting the activity of FABP4 and/or FABP5 may offer a novel strategy for breast cancer therapy.

## Introduction

Despite advancements in diagnosis and treatment, breast cancer remains a leading cause of death among women in the U.S. and worldwide [1]. The global incidence has surged from 641,000 cases in 1980 to over 2.3 million in 2020 [1, 2]. While traditional risk factors such as aging, genetic mutations, family history, and reproductive status play a significant role, obesity has emerged as an additional contributor to the risk of various cancers, including breast cancer [3–5]. However, the molecular mechanisms that link obesity to increased breast cancer risk remain largely unexplained.

Obesity is characterized by dysregulated lipid metabolism, leading to excess lipid accumulation in various organs, tissues, and cells [6]. Given the insoluble nature of lipids in the human body, a family of proteins known as fatty acid binding proteins (FABPs) has evolved to solubilize fatty acids, facilitating their transport and metabolism [7, 8]. The FABP family consists of at least nine members, each with a characteristic tissue distribution, such as liver FABP (L-FABP, FABP1), intestinal FABP (I-FABP, FABP2), heart FABP (H-FABPT, FABP3), adipose FABP (A-FABP, FABP4), epithelial FABP (E-FABP, FABP5), etc. [9, 10]. Since breast tissue primarily consists of adipose tissue, epithelial ducts and lobules, FABP4 and FABP5 are the predominant members involved in coordinating lipid metabolism and maintaining lipid homeostasis in breast tissue [11]. It is of great interest to understand whether and how FABP4 and FABP5 link dysregulated lipid metabolism to breast cancer risk and progression.

Emerging evidence from our group and others has shown that FABP4 and FABP5 play critical roles in breast cancer development and progression in preclinical models [10, 12–15]. For example, obesity induced by a high-fat diet increases the secretion of FABP4 from adipocytes and macrophages into the circulation where soluble FABP4 can directly target breast cancer, inducing ALDH1-mediated breast tumor stemness and invasiveness [16, 17]. In contrast, FABP5 expression in estrogen receptor (ER)-negative cancer cells was shown to promote tumor growth and is associated with a poor prognosis [10, 15]. These findings suggest that FABP4 and FABP5 could serve as important prognostic markers for breast cancer. In this study, we comprehensively analyzed the expression profiles of FABP4 and FABP5 in both normal and breast cancer tissues to assess their clinical significance and potential therapeutic implications.

## Materials and methods

### Study patients

Adjacent normal breast tissues and breast cancer tissues from 96 women were collected at the University of Iowa Hospital and Clinics from 2015 to 2022 with the approval of Institutional Review Board.

### Breast cancer tissue immunohistochemistry

Formalin fixed Paraffin Embedded (FFPE) tissue specimens were deparaffinized in xylene and rehydrated through exposure to graded ethanol solutions. Antigen retrieval was performed by incubating the slides in 10 mM Citrate buffer pH 6.0 at 95 °C for 15 min. Endogenous peroxidase activity was quenched by incubating the slides in BLOXALL Endogenous Blocking Solution (Vector Laboratories, SP-6000) for 10 min at room temperature. Slides were then incubated overnight at 4 °C with primary antibodies, including anti-FABP4 (R&D Systems, AF3150), anti-FABP5 (R&D Systems, AF1476), anti-human vWF (Novus Biologicals, NB600-586) or anti-CD163 (Abcam, ab182422). Depending on the primary antibody source, secondary antibodies were applied using either the anti-goat IgG Polymer Detection Kit (Vector Laboratories, MP-5405) or the anti-rabbit IgG Polymer Detection Kit (Vector Laboratories, MP-7401) for 30 min at room temperature. Color development was achieved using DAB Peroxidase Substrate (Vector Laboratories, SK-4100) or the Vector Red Alkaline Phosphatase Substrate (Vector Laboratories, SK-5100). Slides were counterstained with Hematoxylin (Leica Gill III 3801541) for 10 s at room temperature, and finally mounted with VectaMount^®^ Express Mounting Medium (vector laboratories, H-5700-60).

### Immunohistochemistry slide analysis

Whole section imaging of chromogenic sections was performed using a slide scanner (Leica, Aperio GT 450). Slide scans of all stains can be made available upon request. Scanned tissue sections were loaded into HALO v.3.6.4134.263 (Indica Labs) for quantitative image analysis. The percentage and intensity of FABP4 or FABP5-positive cells in whole-slide tumor tissue was read for H-score calculation. H-score was calculated by the following equation: percentage of weak intensity × 1 + percentage of moderate intensity × 2 + percentage of strong intensity × 3[18]. Quantifications for stromal cells were scored on a scale from 1 to 5, representing the least to the most staining in breast tissues. Percentage of co-expression was estimated using the HALO^®^ Multiplex IHC module.

### Statistical analysis

All data were presented as the mean ± SD unless notified specifically. For statistical analysis, a two-tailed, unpaired student t-test, or two-way ANOVA followed by Bonferroni’s multiple comparison test, were performed using GraphPad Prism 9. Multiple linear regression models were employed to examine the association between FABPs and the clinical parameters as previously described [19]. A statistics test is claimed to be significant if the p-value is less than 0.05.

## Results

### Expression profiles of FABP4 and FABP5 in normal adjacent breast tissue

**Fig. 1:**
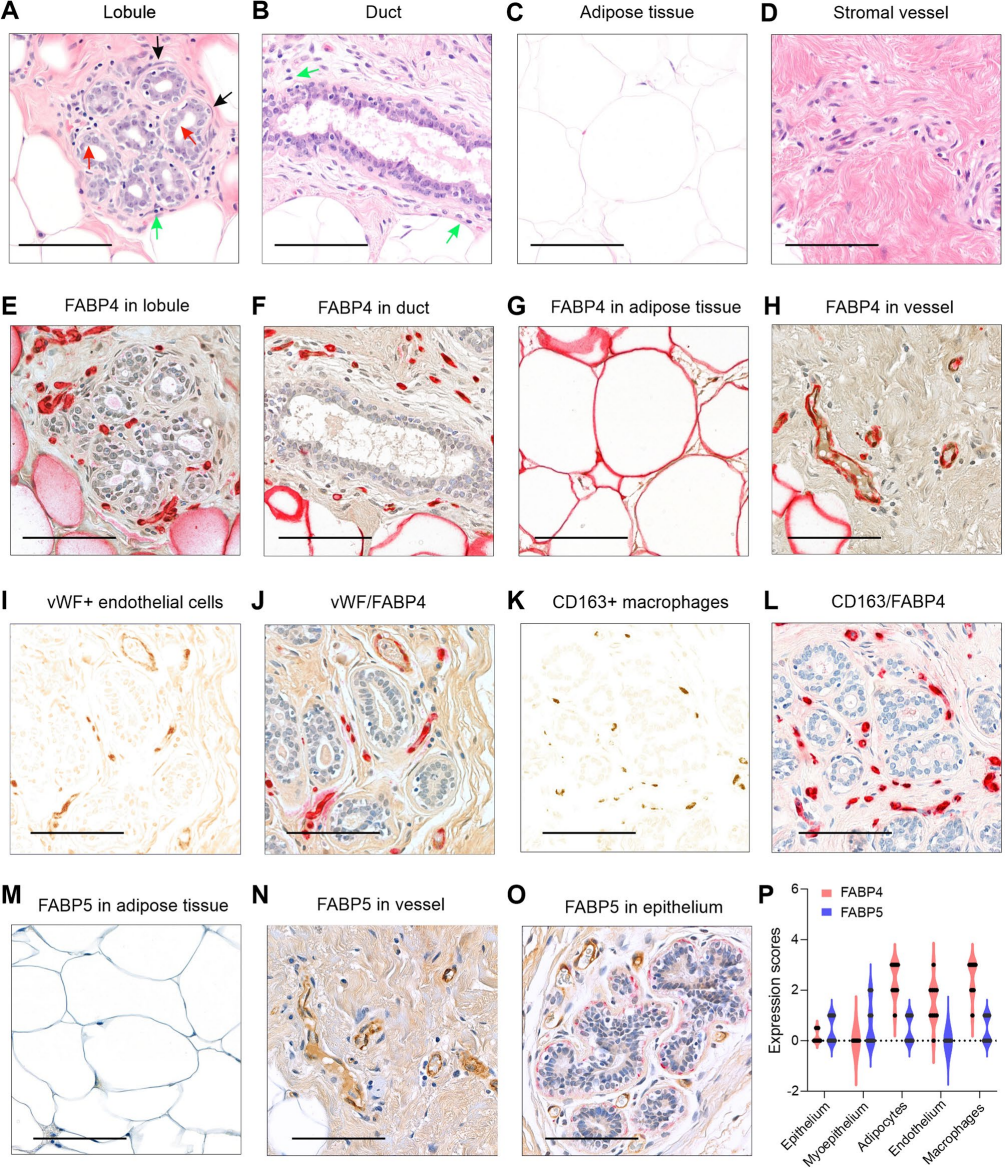
Expression profiles of FABP4 and FABP5 in normal breast tissue. **A**–**D** Representative hematoxylin and eosin (H&E) staining of normal breast tissue, showing lobules (**A**), ducts (**B**), adipocytes (**C**), and blood vessels (**D**). Scale bar: 100 μm. **E**–**H** Representative immunostaining for FABP4 (red) in normal breast tissue, including lobules (**E**), ducts (**F**), adipocytes (**G**), and blood vessels (**H**). Scale bar: 100 μm. **I**, **J** Analysis of colocalization of vWF (brown) (**I**) and FABP4 (red) (**J**) expression in normal breast tissue. Scale bar: 100 μm. **K**, **L** Analysis of colocalization of CD163 (brown) (**K**) and FABP4 (red) (**L**) expression in normal breast tissue. Scale bar: 100 μm. **M**–**O** Representative immunostaining for FABP5 (red) and vWF (brown) in normal breast tissue, showing adipocytes (**M**), blood vessels (**N**), and epithelium (**O**). Scale bar: 100 μm. **P** Comparison of FABP4 and FABP5 expression levels across different cell types in normal breast tissue, including epithelium, myoepithelium, adipocytes, endothelium, and macrophages (n = 9 for each group). The scoring on a 1-to-5 scale was performed for all cells.

To investigate the role of FABP4 and FABP5 in breast cancer development, we first assessed their expression patterns in normal adjacent breast tissue specimens collected from patients with breast cancer. Normal adjacent breast tissue primarily consists of lobules (Fig. 1A), ducts (Fig. 1B), adipose tissue (Fig. 1C), blood vessels (Fig. 1D), and interlobular connective tissue, which provides structural support to the breast and helps maintain its function [20]. Histologically, lobules are mainly composed of milk-producing luminal epithelial cells (red arrows in Fig. 1A) and an outer layer of contractile myoepithelial cells (black arrows in Fig. 1A), which help propel milk from the lobules into the ducts. Similar to lobules, ducts are formed by luminal epithelial cells and myoepithelial cells, serving as a passageway for milk flow from the lobules toward the nipple. Immune cells, such as mononuclear macrophages, are present in both lobules and ducts, which respond to potential invaders and maintain tissue homeostasis (green arrows in Fig. 1A and B).

Immunohistochemical (IHC) staining showed that FABP4 was absent in both luminal epithelial and myoepithelial cells (Fig. 1E, F). Instead, it was highly expressed in the breast stroma, including adipocytes (Fig. 1G) and vessels (Fig. 1H). Using endothelial marker von Willebrand factor (vWF) (Fig. 1I) and macrophage marker CD163 (Fig. 1K), we further demonstrated that FABP4 was highly expressed in vWF^+^ endothelial cells (Fig. 1J) and CD163^+^ macrophages (Fig. 1L). Unlike the stromal cell-specific expression pattern of FABP4, FABP5 was either absent or weakly expressed in the stroma of normal breast tissue, including adipose tissue (Fig. 1M) and vessel endothelium (Fig. 1N). However, FABP5 expression was observed in myoepithelial and epithelial cells in half of the samples (Fig. 1O). The distinct expression patterns of FABP4 and FABP5 in normal breast tissue (Fig. 1P) suggest that FABP4 and FABP5 play different roles in stromal and epithelial cell function.

### FABP4 expression pattern in breast cancer tissue

**Fig. 2:**
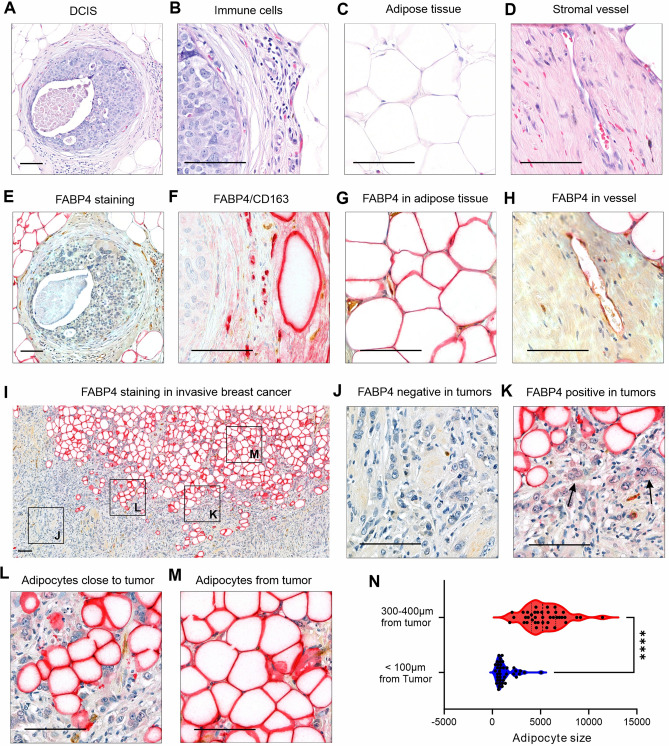
FABP4 expression pattern in breast cancer tissue. **A**–**D** Representative H&E staining of ductal carcinoma in situ (DCIS), including DCIS regions (**A**), surrounding immune cells (**B**), adipocytes (**C**), and stromal vessel (**D**). Scale bars: 100 μm. **E**–**J** Representative immunostaining for FABP4 (red), CD163 (brown) and VWF (brown) in DCIS breast tissue, including DCIS regions (**E**), immune cells (**F**), adipocytes (**G**), and stromal vessels (**H**). Scale bars: 100 μm. **I**–**M** Representative immunostaining for FABP4 in invasive frontline tumor cells and adjacent adipose tissue. Most tumor cells were negative for FABP4 (**J**). Tumor cells close to adipocytes were positive for FABP4 (black arrow) (**K**). Adipocytes adjacent to tumor cells were small (**L**). Adipocytes away from tumor were large (**M**). Scale bar for I: 200 μm, scale bar for panel J-M: 100 μm, (**N**) Comparison of adipocyte sizes at varying distances from tumor cells (p < 0.0001, n = 40 cells).

To investigate the role of FABP4 in breast cancer development and progression, we collected surgical breast cancer tissues from 96 women diagnosed with various subtypes of breast cancer and analyzed FABP expression patterns in these samples (Table 1). In patients diagnosed with ductal carcinoma in situ (DCIS), cancer cells were confined within the milk ducts (Fig. 2A) without spreading into surrounding stromal tissue, including immune cells (Fig. 2B), adipose tissue (Fig. 2C) and vessels (Fig. 2D). FABP4 expression exhibited a similar pattern to that in normal breast tissue: absent in epithelial cells (Fig. 2E), but high in CD163^+^ macrophages (Fig. 2F), adipocytes (Fig. 2G), and vessels (Fig. 2H). In patients with invasive breast cancer, most tumor cells were FABP4-negative (Fig. 2I, J). However, some tumor cells, particularly those near to adipose tissue, exhibited positive staining for FABP4 (Fig. 2K). Among the 96 cases analyzed, 46 cases (47.9%) showed FABP4-positive tumor cells near adipose tissue. Notably, the closer the tumor cells were to adipose tissue, the stronger the FABP4 staining in these cells, suggesting that the ectopic FABP4 staining in these invasive frontline tumor cells may originate from adipose tissue. Indeed, we noticed that the size of adipocytes close to the tumors (< 100 μm,1260 ± 992 µm^2^) was significantly smaller than those farther away from tumors (300–400 μm, 5393.42 ± 2007.39 µm^2^) (Fig. 2L–N). Given that FABP4 secreted by adipose tissue could serve as an adipokine, providing energy support and promoting oncogenic signaling activation in tumor cells [14, 17, 21], these findings provide further evidence that tumor cells could be taking up adipose tissue-derived FABP4 for their invasive benefits at the frontline.

**Table 1:** Summary of patient Information

Clinical parameters		Number of cases (percentage)
Age (years)	30–39	2 (2%)
	40–49	8 (8.3%)
	50–59	18 (18.8%)
	60–69	35 (36.5%)
	70–79	25 (26.3%)
	≥ 80	8 (8.3%)
Vital status	Deceased	15 (15.6%)
	Alived	81 (84.4%)
BMI around diagnosis date	< 18.5 (Underweight)	1 (1%)
	18.5–24.9 (Normal weight)	18 (18.8%)
	25–29.9 (Overweight)	30 (31.3%)
	≥ 30 (Obesity)	47 (49%)
Subtype	DCIS	8 (8.3%)
	Invasive ductal carcinoma	70 (72.9%)
	Invasive lobular carcinoma	9 (9.3%)
	Metaplastic carcinoma	3 (3.2%)
	Other	6 (6.3%)
Tumor size	< 2 cm	56 (58.3%)
	2-5 cm	33 (34.4%)
	> 5 cm	7 (7.3%)
Elston Ellis grade	Grade 1	20 (20.8%)
	Grade 2	40 (41.7%)
	Grade 3	30 (31.3%)
	Other	6 (6.25%)
Lymphovascular invasion status	Presence	22 (22.9%)
	Absence	74 (77.1%)
Metastasis status	No metastasis	63 (65.6%)
	Lymph node metastasis	26 (27.1%)
	Distant metastasis	7 (7.3%)

### FABP5 expression pattern in breast cancer tissue

**Fig. 3:**
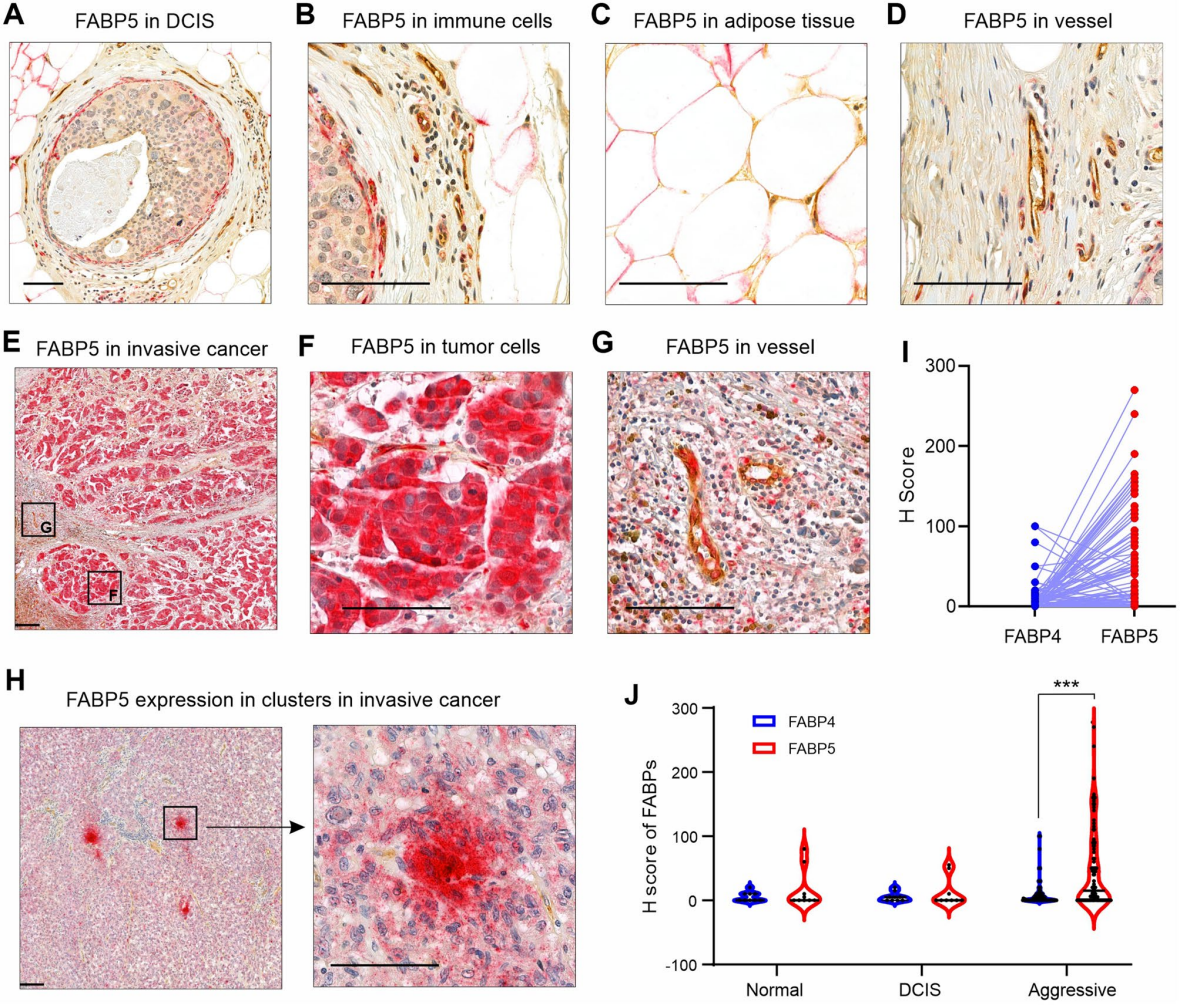
FABP5 expression pattern in breast cancer tissue. **A**–**D** Representative immunostaining for FABP5 (red) and vWF (brown) in DCIS, including DCIS regions (**A**), surrounding immune cells (**B**), adipocytes (**C**), and stromal vessel (**D**). Scale bars: 100 μm. **E**–**H** Representative immunostaining for FABP5 (red) and vWF (brown) in invasive breast cancer, showing two distinct patterns of FABP5 expression in tumor cells: ubiquitous expression in tumor cells (**E**, **F**) or clusters of positive cells sporadically distributed throughout the tumor tissue (**H**). Scale bars: 100 μm. **I** Correlation analysis between FABP4 and FABP5 expression levels in tumor cells of patients with breast cancer (n = 96). **J** Comparison of FABP4 and FABP5 expression levels across normal breast tissue, DCIS, and aggressive cancer (p < 0.001, n = 96)

Next, we analyzed FABP5 expression pattern in the same cohort. Similar to our observations in normal breast tissue, DCIS tissues showed weak FABP5 expression in epithelial tumor cells and myoepithelial cells (Fig. 3A), CD163^+^ macrophages (Fig. 3B), adipocytes (Fig. 3C) and vessels (Fig. 3D). However, invasive breast cancer tissues exhibited a significant increase in FABP5 expression (Fig. 3E), mainly in tumor cells (Fig. 3F). Vessel endothelial cells also exhibited increased expression of FABP5 (Fig. 3G). In contrast to FABP4, which was primarily expressed in tumors adjacent to adipose tissue (Fig. 2K), FABP5 expression in tumor cells (66 out of 96 cases) was either ubiquitously (68.2%, 45 out of 66 cases) (Fig. 3E) or clustered (31.8%, 21 out of 66 cases) in sporadically distributed groups of cells throughout the tumor (Fig. 3H). Statistical correlation analysis showed that there were no correlations between the expression pattern of FABP4 and FABP5 in tumor cells (Fig. 3I). In addition, in tumor cells overall expression of FABP5 (47.2 ± 60.46) was significantly higher than that of FABP4 (8.49 ± 18.24) (Fig. 3J). Altogether, the different expression patterns/levels of FABP4 and FABP5 in invasive breast cancer suggested their distinct roles in breast cancer development and progression.

### FABP4 expression at the tumor border correlates with lymphovascular invasion

**Fig. 4:**
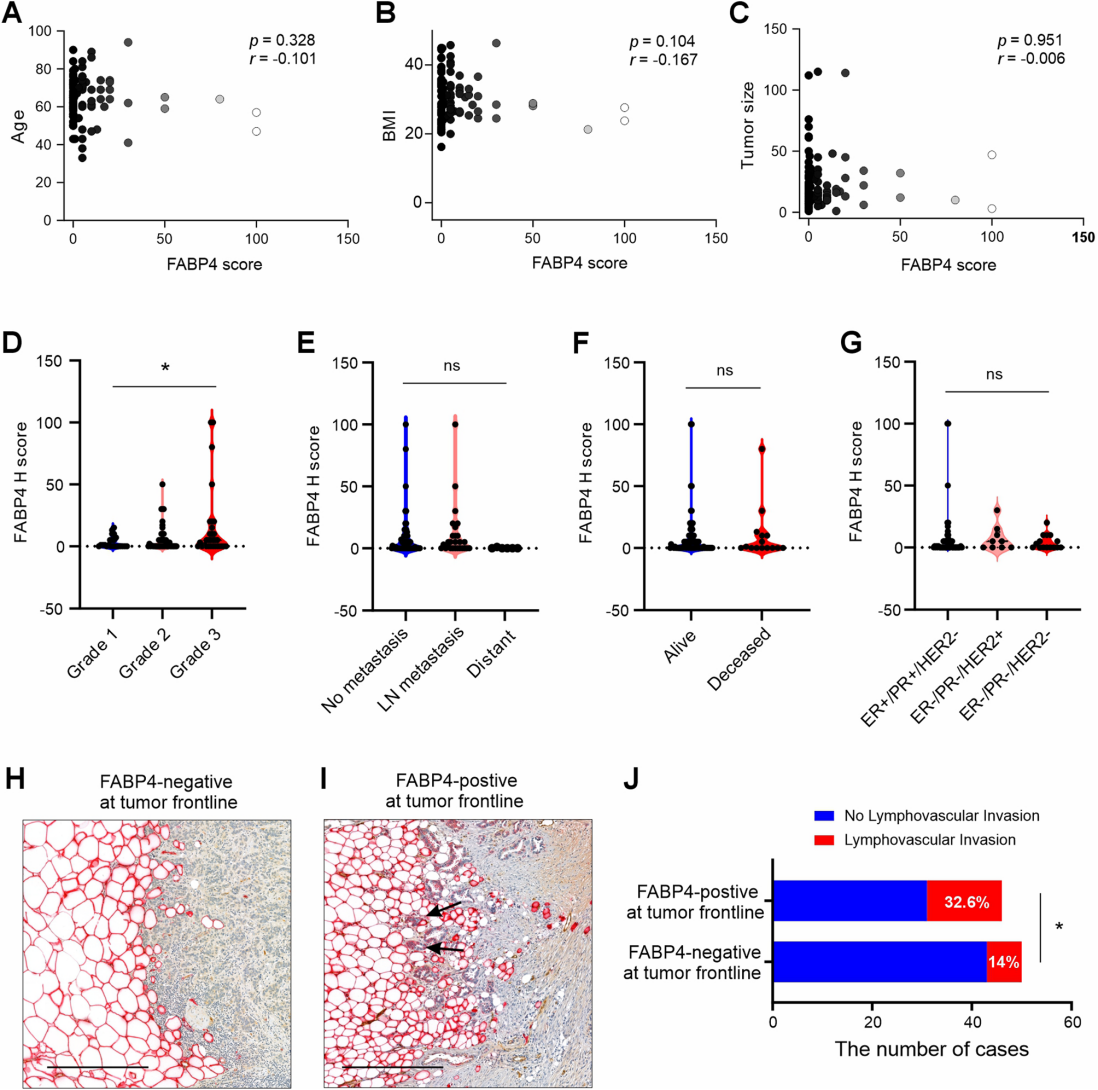
Correlations of FABP4 expression in tumor cells with clinical parameters. **A**–**C** Correlation analysis between FABP4 expression in tumor cells and various clinical parameters, including age (**A**), BMI (**B**), and tumor size (**C**). **D**–**F** Comparison of FABP4 expression levels in tumor cells of breast cancer patients with different tumor grade (**D**), metastasis status (**E**), and patient vital status (**F**) (*p < 0.05, ns: non-significant). **G** FABP4 expression across different breast cancer subtypes, including ER + /PR + /HER2-, ER-/PR-/HER2 + , and ER-/PR-/HER2- (ns: non-significant). **H**, **I** Representative immunostaining for FABP4 in invasive frontline tumor cells and adjacent adipose tissue. No FABP4 was expressed in tumor cells (**H**) and FABP4 was expressed in tumor cells close to adipocytes (black arrows) (**I**). Scale bars: 100 μm. **J** Comparison of lymphovascular invasion percentage between patients with FABP4-positive (n = 46) and patients with FABP4-negative (n = 50) staining at the tumor front line (*p < 0.05)

To dissect the potential role of FABP4 in breast cancer development and progression, we first analyzed the correlation of FABP4 expression in tumor cells with various clinical parameters, including age (Fig. 4A), BMI (Fig. 4B), tumor size (Fig. 4C), Elston-Ellis grade (Fig. 4D), metastasis (Fig. 4E), and patient vital status (Fig. 4F). None of these clinical parameters showed a significant correlation with FABP4 expression in tumors except the Elston-Ellis grade (Fig. 4D), which showed a weak but significant correlation with FABP4 H-score (Grade 1: 3.22 ± 4.69; Grade 2: 6.27 ± 10.99; Grade 3: 15.13 ± 28.1) in tumor cells. We further assessed FABP4 expression across different breast cancer subtypes, including hormone sensitive (ER + /PR + /HER2-), HER2 positive (ER-/PR-/HER2 +), and triple negative (ER-/PR-/HER2-). The overall expression levels of FABP4 in tumor cells were very low across breast cancer subtypes, and there were no significant differences in FABP4 expression H-score among various subtypes (Fig. 4G), suggesting minimal impact of hormones (*e.g.* estrogens, progesterone) or growth factor signals (HER2) on FABP4 expression. As FABP4-positive tumor cells were mainly located in regions that often interacted with adipose tissue (Fig. 2K), we further determined whether FABP4 expression at the tumor/adipose tissue border (Fig. 4H, I) impacted clinical parameters. Interestingly, among patients with FABP4 expression at the tumor border, 32.6% (15 out of 46 cases) exhibited tumor lymphovascular invasion, a significantly higher rate than the 14% (7 out of 50 cases) observed in patients without FABP4 expression at the tumor border (Fig. 4J). Since tumor cells mainly spread through the lymphatic and vascular systems [22], FABP4 may mediate adipose/tumor crosstalk by enhancing tumor invasion.

### FABP5 expression in tumor cells is associated with tumor metastasis

**Fig. 5:**
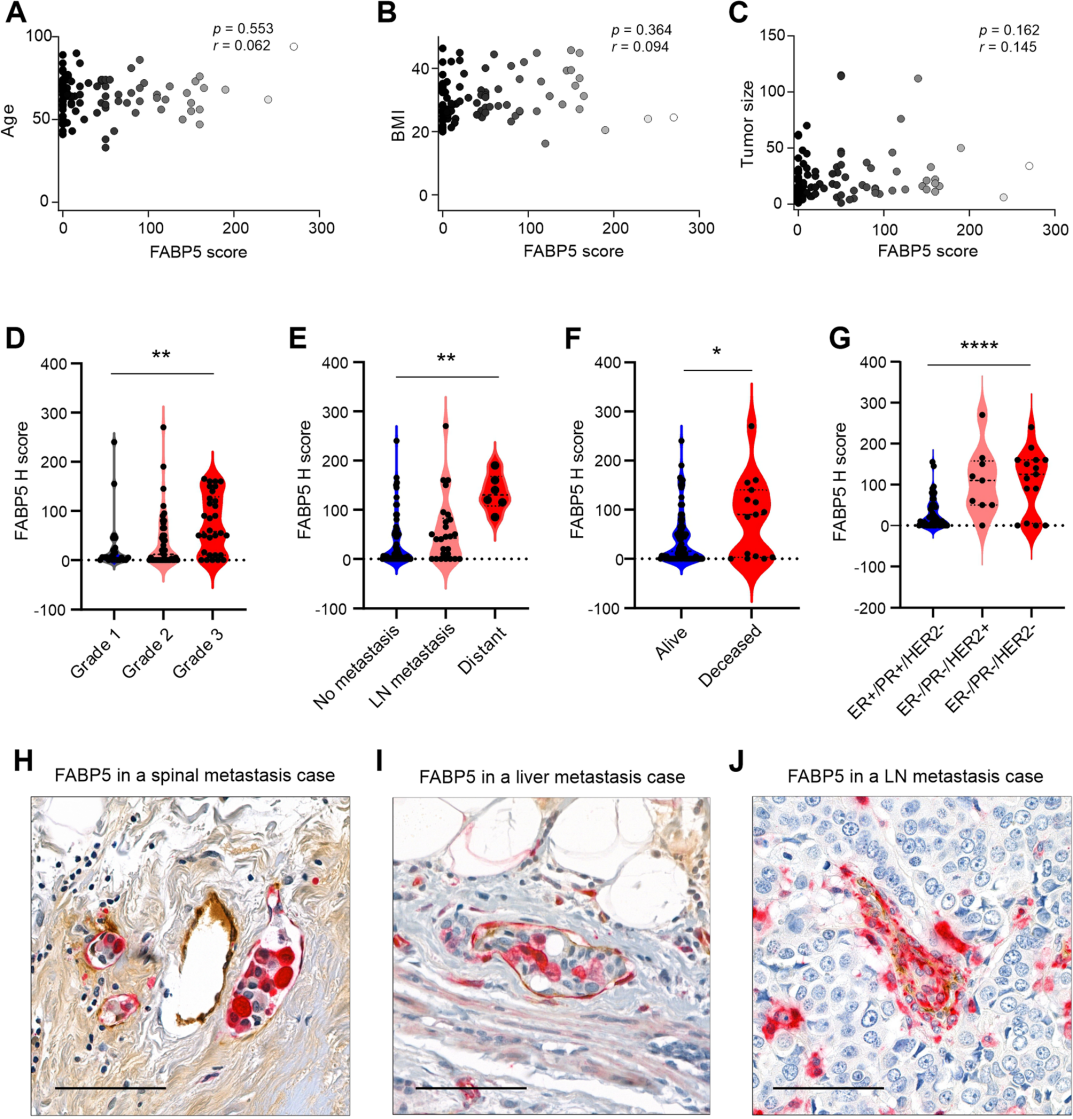
Correlations of FABP5 expression in tumor cells with clinical parameters. **A**–**C** Correlation analysis between FABP5 expression in tumor cells and various clinical parameters, including age (**A**), BMI (**B**), and tumor size (**C**). **D**–**F** Comparison of FABP5 expression levels in tumor cells of breast cancer patients with different tumor grade (**D**), metastasis status (**E**), and patient vital status (**F**) (*p < 0.01, **p < 0.01). **G** FABP5 expression across different breast cancer subtypes, including ER + /PR + /HER2-, ER-/PR-/HER2 + , and ER-/PR-/HER2- (****p < 0.0001). **H, I, J** Representative images showing strong FABP5 expression in metastatic tumor cells within blood vessels in patients with spinal metastasis (**H**), liver metastasis (**I**) and lymph node (LN) metastasis (**J**)

To further investigate the role of FABP5 in invasive breast cancer, we first analyzed the correlation between FABP5 expression in tumor cells and various clinical parameters, including age (Fig. 5A), BMI (Fig. 5B), tumor size (Fig. 5C), tumor Elston-Ellis grade (Fig. 5D), metastasis (Fig. 5E) and patient vital status (Fig. 5F). Similar to FABP4, FABP5 expression did not correlate with patient age, BMI and tumor size (Fig. 5A–C). However, FABP5 expression significantly corelated with tumor grade, tumor metastasis and patient vital status (Fig. 5D–F). Further analysis of FABP5 expression in tumor cells across different breast cancer subtypes demonstrated that both triple negative (108.33 ± 76.36, H-score) and HER2^+^ (108.33 ± 80.70, H-score) subtypes exhibited significantly higher levels of FABP5 expression (p < 0.0001) compared to the hormone sensitive subtype (29.76 ± 37.41, H-score) (Fig. 5G). Notably, in multiple specimens with tumor metastases, we observed that tumor cells within blood vessels in the primary tumors exhibited strong expression of FABP5 expression (Fig. 5H–J), suggesting a possible pro-metastatic activity of FABP5 in tumor cells.

## Discussion

Breast epithelium, embedded in adipose tissue, is significantly influenced by lipid metabolism during both normal differentiation and carcinogenesis [23]. In breast cancer, fatty acid levels are generally higher than in normal tissue [24]. Cancer cells in the breast acquire lipids either from exogenous sources, such as surrounding adipose tissues or through their own de novo lipogenesis [25]. However, targeting lipids directly for therapeutic purposes is challenging because of the complexity of lipid species and the compensatory mechanisms within lipid synthesis pathways. Rather than focusing on lipids themselves, inhibiting the activity of FABPs, which act as lipid chaperones, may offer a novel strategy for treatment of lipophilic breast cancer.

In normal breast tissue, FABP4 is predominantly expressed in stromal cells, including adipocytes, endothelial cells, and macrophages, while FABP5 exhibits a weak expression in myoepithelial and epithelial cells. Given these distinct expression patterns, FABP4 likely plays a key role in supporting lipid metabolism and functions in stromal cells, whereas FABP5 may contribute to epithelial cell activity in normal breast tissue. These differences suggest complementary roles for stromal and epithelial cells in coordinating lipid transport and metabolism within the breast microenvironment.

Interestingly, in breast cancer tissue, we further observed that FABP4 remains primarily expressed in stromal adipocytes, endothelial cells and macrophages, whereas FABP5 is significantly upregulated in epithelial-derived tumor cells. Our findings highlight several key insights: (1) Epithelial-derived breast cancer cells typically do not express FABP4. However, invasive tumor cells near adipose tissue often express FABP4, suggesting that its presence in tumor cells may be ectopically acquired from neighboring adipocytes. This is supported by our observation of reduced adipocyte size at the tumor front, consistent with findings from other animal models [26]. These data suggest that FABP4 facilitates the transport of exogenous fatty acids from the stroma to tumor cells, activating oncogenic signaling and enhancing lipid responses for breast cancer progression. (2) In aggressive breast cancers, especially ER-negative subtypes, FABP5 is significantly upregulated in tumor cells compared to normal tissue or DCIS. Elevated FABP5 expression is positively correlated with tumor metastasis and decreased patient survival, suggesting that FABP5 functions as an intrinsic lipid chaperone, promoting lipid metabolism and tumor dissemination. This aligns with previous studies showing that FABP5 expression is associated with poor survival in TNBC during retinoic acid therapy [10]. Our studies further support a pro-metastatic role for FABP5 in breast cancer progression. Given the established link between obesity-associated lipid dysregulation increased breast cancer risk [27, 28], FABP4 and FABP5 play critical roles in facilitating fatty acid oxidation and metabolism, as well as promoting oncogenic lipid signaling pathways. These characteristics position them as unique markers of aggressive breast cancer.

In summary, while FABP4 and FABP5 play distinct roles in regulating lipid availability for tumor cells, both are associated with poor prognoses in breast cancer. Given our recent development of multiple anti-FABP4 antibodies, which showed significant efficacy in reducing mammary tumor growth and metastasis in animal models [29], FABP4 and FABP5 represent promising therapeutic targets, and blocking their activity may offer novel strategies for breast cancer treatment.

## Data Availability

Data is provided within the manuscript or supplementary information files.
